# Metabolic Changes After the Implementation of a Recreational Physical Activity Program at Mexican Elderly Adults’ Welfare Homes

**DOI:** 10.3390/geriatrics11030057

**Published:** 2026-05-07

**Authors:** Moisés Martínez Briseño, Manuel Abraham Gómez-Martínez, Diana Rodríguez-Vera, Kenneth Rubio Carrasco, Raúl Lugo Villegas, María de los Ángeles Frías Fernández, Marco A. Loza-Mejía, José A. Morales-González, Rodolfo Pinto-Almazán, Etzel Cruz Cruz, Arely Vergara-Castañeda

**Affiliations:** 1 DIF Unidad Central, Huixquilucan de Degollado, Mexico City 52760, Mexico; moisesmartinezbriseo@hotmail.com; 2 Chemical Sciences School, Universidad La Salle-México, Benjamín Franklin 45, Mexico City 06140, Mexico; krc@quimica.unam.mx (K.R.C.); raul.lugo@lasalle.mx (R.L.V.); 3 Department of Public Health, Faculty of Medicine, Universidad Nacional Autónoma de México, Mexico City 04510, Mexico; mgomez@facmed.unam.mx; 4 Postgraduate Studies and Research Section, Escuela Superior de Medicina del Instituto Politécnico Nacional, Plan de San Luis y Diaz Mirón s/n, Mexico City 11340, Mexico; drodriguezv1904@alumno.ipn.mx (D.R.-V.); rodolfopintoalmazan@gmail.com (R.P.-A.); 5 Facultad de Química, Universidad Nacional Autónoma de México, Mexico City 04510, Mexico; 6 Programa Jóvenes a la Investigación en Matemáticas y Ciencias Experimentales, Escuela Nacional Colegio de Ciencias y Humanidades Plantel Azcapotzalco, Universidad Nacional Autónoma de México, Mexico City 02420, Mexico; mariadelosangeles.frias@cch.unam.mx; 7 Design, Isolation, and Synthesis of Bioactive Molecules Research Group, Universidad La Salle-México, Benjamín Franklin 45, Mexico City 06140, Mexico; marcoantonio.loza@lasalle.mx; 8 Laboratorio de Medicina de Conservación, Escuela Superior de Medicina del Instituto Politécnico Nacional, Plan de San Luis y Diaz Mirón s/n, Mexico City 11340, Mexico; jmorales101@yahoo.com.mx; 9 Dirección de Posgrado e Investigación, Universidad La Salle Oaxaca, Santa Cruz Xoxocotlán, Oaxaca 71230, Mexico; etzel.cruz@ulsaoaxaca.edu.mx; 10 Research Group on Development and Innovation in Health and Nutrition Promotion and Education, Universidad La Salle-México, Benjamín Franklin 45, Mexico City 06140, Mexico

**Keywords:** aging, metabolic control, physical activity intervention, blood pressure control, active and healthy ageing

## Abstract

**Background/objective:** Hypertension and type 2 diabetes are major causes of morbidity in older adults. Although pharmacological treatments remain the cornerstone of management, structured physical activity has been shown to provide additional benefits, yet evidence from institutionalized populations in Latin America is limited. This study evaluated the impact of a 12-month supervised exercise program on blood pressure (BP), glycated hemoglobin (HbA1c), and body composition in elderly people attending welfare homes in Mexico. **Methods**: A community-based intervention trial was conducted (February 2018–January 2019) with 260 adults (aged > 60 years) with hypertension and/or diabetes. Participants were allocated based on shelter site to either a control group (n = 129; pharmacological treatment only) or an intervention group (n = 131; pharmacological treatment plus five one-hour supervised recreational physical activity sessions per week). Monthly anthropometric, clinical, and biochemical measurements were analyzed using parametric/non-parametric tests and estimation of effect size (Cohen’s d). **Results**: Median age was 70 years (86% female). After 12 months, systolic BP decreased from 148.4 to 129.7 mmHg in the intervention group vs. 147.7 to 131.3 mmHg in controls. Diastolic BP showed greater reduction in the intervention group (−25%; 93.1 to 68.9 mmHg) than in controls (−13.5%; 88.1 to 76.2 mmHg). HbA1c reductions were also superior in the intervention group (–2.28% vs. –1.86%). Both groups lost fat mass, but lean mass preservation was limited. **Conclusions:** Structured community-based physical activity significantly improves BP, glycemic control, and body composition, supporting its integration into routine institutional care with limited resources.

## 1. Introduction

The global population is aging at an unprecedented pace, with the proportion of individuals over 60 years expected to nearly double between 2015 and 2050 [1]. In Mexico, demographic projections estimate that adults ≥ 60 years will represent more than 25% of the population by 2050 [2,3]. This demographic shift is accompanied by a growing burden of chronic degenerative diseases, particularly hypertension and type 2 diabetes, which together account for more than 40% of mortality in older adults [4].

Hypertension is one of the most prevalent chronic conditions worldwide and a major risk factor for cardiovascular and renal complications [5]. Since its prevalence increases with age, especially in rapidly aging societies such as Mexico, the prevention and management of hypertension represent a critical public health priority [6,7,8,9,10]. Recent national surveys in Mexico estimate that 24% of adults are hypertensive, with a high proportion remaining undiagnosed or uncontrolled, particularly among older adults [11,12,13]. Furthermore, the RIHTA registry documented that up to 60% of hypertensive patients remain uncontrolled despite treatment, highlighting the urgent need for complementary interventions [14].

Similarly, diabetes mellitus is strongly linked to sedentary lifestyles and obesity, both of which are increasingly common in urbanized and low-resource settings. In Mexico, higher levels of leisure-time physical activity are associated with lower risks of abdominal and general obesity [15]. Across Latin America, urban built environments—such as less fragmented and greener areas—are associated with lower body mass index (BMI) and reduced obesity and type 2 diabetes prevalence [16].

Lifestyle interventions, particularly structured physical activity, have emerged as effective therapeutic strategies for improving cardiometabolic outcomes in older adults. Recent guidelines from the European Society of Hypertension and the American Diabetes Association emphasize that aerobic and resistance training can reduce blood pressure and HbA1c to a similar extent as first-line pharmacological therapies, even in patients already receiving drug treatment [17,18]. Furthermore, multimodal approaches that integrate exercise and behavioural counselling have demostrated superior long-term benefits for community-dwelling seniors [19,20].

Despite this evidence, however, limited research has been conducted on the feasibility and effectiveness of recreational physical activity interventions in community or social assistance centers serving elderly populations in Latin America, where socioeconomic vulnerability, polypharmacy, and limited healthcare resources present further challenges. Government-supported welfare homes in particular represent a unique opportunity to implement structured, recreational and low-cost physical activity programmes on a large scale.

Therefore, this study aimed to evaluate the effects of a 12-month supervised recreational physical activity programme on blood pressure, glycemic control, and body composition in elderly adults attending to welfare homes rum by the National System for the Integral Development of Families (SNDIF), also known to as DIF Nacional or simply DIF, in comparison with standard pharmacological treatment alone offered by the same social welfare institution.

## 2. Materials and Methods

### 2.1. Study Design and Setting

Between February 2018 and January 2019, we conducted a community-based intervention trial which included adults aged 60 years and over with hypertension and/or type 2 diabetes. These adults attended two shelters from the National System for the Integral Development of Families (SNDIF). The shelters were based in Huixquilucan, Mexico. These shelters are located in vulnerable areas and are characterized by a high level of commitment and active participation of unprotected individuals and communities in the programmes and services offered by the institutions.

### 2.2. Participants and Eligibility Criteria

Both, man and women aged over 60 years old with previous diagnostic of hypertension and diabetes were enrolled, pharmacological treatment for those elderly people was linagliptin (5 mg) and telmisartan-hydrochlorothiazide (80 mg/12.5 mg), according to the management of the medical area from these shelters. Also, there were only considered the ones who had a minimum of two medical consultations within three months previous to the beginning of the program and the ones that agreed to participate in the program and signed the consent form. Participant eligibility was assessed during a pre-enrollment screening phase based on clinical history, exclusion criteria considered elder people who presented any physical, legal, or mental disability that would keep them from performing any kind of activities, obesity (>40 kg/m^2^) and resting blood pressure (BP). Individuals with BP overs 140/90 mmHg during screening were excluded to ensure pharmacological stability and patient safety. It is important to note that baseline values reported in this study (recorded on the first day of intervention) may show slight deviations from screening thresholds due to inherent physiological variability and the ‘white coat effect’ common in institutionalized settings [21]. Also, those who had a confirmed diagnosis of kidney and liver failure, hyperthyroidism and hypothyroidism, and intestine bad absorption, those who had gone through surgery within the last two years, and finally all with a heart attack background or any kind of drug or substance abuse were stepped aside this study nor the activities of the welfare homes. Elimination criteria included, all participants who had less than 80% attendance to designated activities, those who couldn’t keep up with the continuance of the program, or subjects who quit the program or withdrew the consent form.

### 2.3. Allocation and Intervention

As a community-based intervention design was adopted, participants were allocated at the cluster level (shelter site) rather than through individual randomization. This approach was selected to prevent inter-participant contamination, as the shared living environment and social interactions within the welfare homes would have inevitably exposed the control group to the intervention’s components. Furthermore, a cluster-based assignment ensures intervention fidelity and logistical feasibility, maintaining social cohesion among residents while allowing for the effective implementation of supervised physical activity within the institutional infrastructure.

The intervention group received besides standard pharmacological therapy plus a structured, supervised multicomponent recreational physical activity program, during 12 months.

The supervised exercise program consisted of 60-min sessions, five times per week (Monday to Friday), according exercise recommendations in older adults [22]. The daily intervention was divided into three distinct phases and began with a 10-min multi-component warm-up designed to enhance joint mobility and neuromuscular activation. Participants first performed joint mobility exercises in a cephalo-caudal sequence, including gentle neck rotations, shoulder circles, and ‘apple picking’ movements, followed by wrist and ankle rotations. This was followed by gentle dynamic activation to gradually increase heart rate, featuring marching in place with rhythmic arm swinging, side-to-side steps with coordinated clapping, and heels-to-buttocks flexions using chair support for stability. To conclude the preparation, sensory awakening and balance activities were implemented, including controlled lateral weight transfers, alternating toe-heel, and ‘the hug’ stretch to expand rib cage capacity and optimize respiratory function. All movements were performed at a low-to-moderate intensity to ensure safety and prepare participants for the main exercise session.

The intervention’s main phase consisted of a 40-min multicomponent circuit divided into two functional blocks: muscle strength focused on functional independence through simulated daily activities, including chair stands (‘throne lifts’), shoulder and back strengthening using resistance bands (‘cupboard reaching’), and partner-based isometric leg presses with soft balls, and the cardiovascular resistance one aimed to impact metabolic health through low-impact aerobic activities, such as obstacle-navigation circuits (‘messenger’s step’) and coordinated rhythmic dancing to age-appropriate music.

To prevent monotony and enhance agility, recreational activities were integrated, including ‘balloon volleyball’ to stimulate safe, explosive limb extensions, and ‘target tossing’ with hoops or sponge balls to improve upper-body thrust and precision. Additionally, ‘mirroring’ exercises in pairs were employed to foster social interaction and observational skills through the imitation of strength movements. These playful elements ensured high engagement while maintaining a moderate training intensity throughout the sessions.

Sessions concluded with a 10-min cool-down phase aimed at facilitating cardiovascular recovery and promoting muscle relaxation. This stage involved low-intensity rhythmic movements (slow walking), followed by static stretching of major muscle groups (held for 20–30 s each) to enhance flexibility. To optimize recovery, diaphragmatic breathing exercises and progressive relaxation techniques were implemented. Finally, a subjective recovery assessment was conducted to ensure participants returned to their physiological baseline before moving on to other activities scheduled within the centers.

All activities were designed to adapt to the participants’ conditions and could be carried out standing, sitting, or with breaks. The control group continued just with pharmacological treatment alone without changes on the type but some adjustments based on the management of their conditions in both groups.

### 2.4. Data Collection

Measurements included systolic blood pressure (SBP) and diastolic blood pressure (DBP) were measured using a Welch Allyn sphygmomanometer (Welch Allyn, Hillrom, NY, USA) and classified according AHA guidelines, glycated hemoglobin (HbA1c) (HPLC Bio-Rad D-10 (HPLC Glycosylated Hemoglobin Analyzer (D-10 System; Bio-Rad Laboratories, Inc. Hercules, CA, USA)), and body composition using a segmental body composition monitor (FitScan BC-601F, Tanita^®^, Tokyo, Japan), BMI was calculated as weight in kilograms divided by height in meters squared (kg/m^2^). Data were collected monthly by trained staff.

### 2.5. Statistical Analysis

Analyses used parametric/nonparametric group comparisons, with Shapiro-Wilk test for data distribution, a comprising qualitative and quantitative analysis for independent groups such as the paired *t*-test or, on the other hand, the Friedman test for repeated tests with nonparametric distribution and Cohen’s d for effect size d = $\frac{M1-M2}{SD\;pooled}$. A *p* < 0.05 was considered statistically significant. All statistical analyses were performed using IBM SPSS Statistics, Version 21, IBM Corporation, Armonk, NY, USA.

### 2.6. Ethical Considerations

Ethics approval was obtained from the Faculty of Medicine Ethics Committee, Universidad La Salle (CIE-2018-1, on 12 February 2018).

## 3. Results

### 3.1. Baseline Characteristics

From a cohort of 264 elderly people (who gave us their consent for participating), 4 of them were eliminated because of incomplete data information and excluded from the analysis. All participants were assigned to two different groups: 129 were from the intervention group and 131 from the control group. In total, 224 females (86.2%) and 36 males (13.8%). The median age was 70.0. Regarding basal characteristics, all are described in Table 1, in which both groups were comparable regarding the lean and fat composition of participants’ variables; on the other hand, a greater weight was observed in the intervention group at the beginning, but also a lower age average. There were statistically significant differences from the intervention group members as well, who presented higher diastolic blood pressure and glycated hemoglobin.

### 3.2. Follow-Up Results

After the six-month measurements, the intervention group presented higher decreases in diastolic blood pressure in comparison with the control group (16.92 vs. 14.76 mmHg) and glycated hemoglobin (1.86 vs. 1.17%).

**Figure 1:**
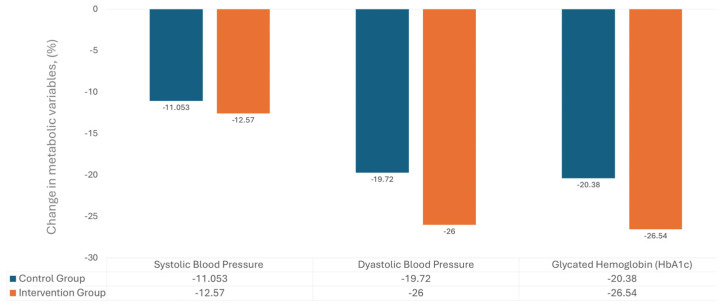
One-year changes in blood pressure and HbA1c: A comparison between control and intervention groups.

Furthermore, after twelve months of the implementation of the program, the intervention group presented a statistically significant improvement, decreasing in all metabolic measurements compared with the control group: weight (3.73 vs. 2.99; Cohen’s D 2.53 vs. 1.86), body mass index (1.55 vs. 1.38; Cohen’s D 2.36 vs. 1.84) (Figure 1).

**Figure 2:**
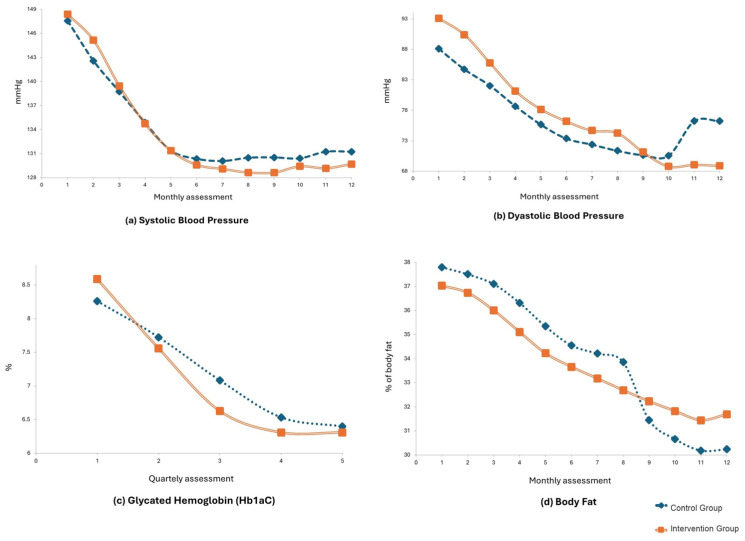
Changes in blood pressure and glycated hemoglobin in the control and intervention groups after a one-year follow-up. Assessment 1 correspond to baseline.

All results reported in Figure 2 showed a statistical difference between the groups’ measurements (*p* < 0.001) for the time the program took part (12 months), considering a Friedman test. The monitoring measurements (fat mass percentage, glycated hemoglobin, diastolic blood pressure, and systolic blood pressure) suggest significant differences (statistical significance *p* ˂ 0.001) regardless of the group they belonged to, among the period of one-year measurements.

The graphic presented for the systolic blood pressure measurements shows a significant decrease between the first five months’ values: control group 147.56 vs. 131.40 mmHg (difference between baseline and 5 month’s measurement 16.16 mmHg; Cohen’s D 2.42; *p* < 0.001); intervention group 148.36 vs. 131.39 mmHg (16.97 mmHg; Cohen’s 2.88; *p* < 0.001); nevertheless, starting the sixth month of the evaluation program, a tendency of maintenance was presented for both groups: control group 130.37–131.26 mmHg (difference between 5-month and 12 month’s measurement −0.89 mmHg; Cohen’s D −1.76); intervention groups 129.61–129.71 mmHg (−0.1 mmHg; Cohen’s D −0.41). On the other hand, when comparing values between baseline and 12 month’s a decrease in the control group from 147.65 to 131.26 mmHg; Cohen’s D 1.70 and in the intervention group from 148.36 to 29.71 mmHg; Cohen’s D 2.46 CI_95%_ (2.14–2.78), was observed.

Results presented in the diastolic blood pressure graphic also showed a significant decrease during the first nine-month evaluations (control group 88.10 vs. 70.62 mmHg; Cohen’s D 2.57; intervention group 93.08 vs. 71.12 mmHg; 5.33; Cohen’s D 5.33), but after the ninth month, both group measurements showed an increment of blood pressure (control group −5.67 mmHg and intervention group −0.10), and they crossed each other between the 9th and 10th month. The control group, which presented continuously lower blood pressure numbers, finished with a higher diastolic blood pressure mean (control group 76.20 mmHg vs. intervention group 68.88 mmHg; Cohen’s D 0.19 vs. 5.78).

The glycated hemoglobin graphic presents a decreasing tendency line for both groups, at 9th month (control group 1.724% after; intervention group 2.27%). Both lines crossed each other between the first and the third month evaluation (7.8%), which resulted in a decreasing glycated hemoglobin percentage mean after the final evaluation (control group 1.86% vs. intervention group 2.28%; Cohen’s D 6.53).

For the fat body composition comparison graphics, it was observed a constant decrease for both groups from the baseline data collected up to the eleven-month measure (control group 37.80 vs. 30.24%; Cohen’s D 2.86; intervention group 37.02 vs. 31.69%; Cohen’s D 1.192), even though the intervention group means remained lower than the ones of the control group. After the ninth month evaluation, the control group measurements get lower than the ones of the intervention group.

Respected to the percentage of lean mass at the end of this study, the control groups got a minor gain compared to the intervention group (−0.97% vs. −3.47%; Cohen’s D 0.300 vs. 2.40).

**(a)**  **Systolic Blood Pressure:** Both groups show a significant initial reduction, stabilizing after the fifth month. It is observed that the intervention group (solid line) maintains levels slightly lower than those of the control group toward the end of the study, remaining below 130 mmHg.
**(b)**  **Dyastolic Blood Pressure:** This figure shows a sustained downward trend in Diastolic Blood Pressure for both groups during the first 10 months. A rebound in levels is notable in the control group after month 10, while the intervention group maintains the downward trend, ending the study period with values close to 69 mmHg.
**(c)**  **Glycated Hemoglobin**: The graph illustrates the quarterly variation in the percentage of HbA1c. Although both groups started with values above 8%, the intervention group showed a steeper downward trend beginning in the second quarter, achieving stabilization near 6.3% compared to a more moderate reduction in the control group, suggesting an optimization of long-term metabolic control compared to the control group’s response.
**(d)**  **Body Fat**. The composition of body fat over 12 months is detailed. It is observed that the control group experienced an accelerated decline between months 8 and 11. However, the intervention group shows a more consistent and progressive reduction throughout most of the year, ending the study in the 31–32% range.

## 4. Discussion

This study was aligned with the approach to healthy aging, advocating that future care for older people should aim to maintain functional capacity and control of their diseases, enabling well-being in old age [23,24].

Demonstrates that a structured, shelter-based physical activity program significantly improves blood pressure and glycemic control in elderly adults with hypertension and type 2 diabetes, even when participants are already receiving pharmacological treatment. The observed reductions in systolic and diastolic blood pressure (~18 and ~25 mmHg, respectively) and HbA1c (−2.28%) are clinically meaningful [25], comparable to or greater than those reported in meta-analyses of exercise interventions in older populations [26].

Our findings gain further relevance in the Mexican context, where recent epidemiological studies show persistently high prevalence of uncontrolled hypertension among older adults [27]. The reduction in HbA1c observed in this study also addresses the unmet need for effective strategies in settings where diabetes prevalence continues to rise in tandem with obesity and physical inactivity [28,29].

Importantly, community-based exercise programs similar to ours have shown consistent benefits in mobility, cardiovascular outcomes, and quality of life in older adults [26]. For example, reviews of group-based interventions combining physical activity and nutrition confirm improvements in functional status and mobility [30]. The recent reviews demonstrated that even hybrid models (in-person plus remote support) can improve strength and functional performance in frail seniors [30,31]. These findings strengthen the argument that scalable, low-cost exercise interventions are feasible and effective in institutional and community settings.

While body fat reduction was significant in both groups, lean mass declined slightly, particularly in the intervention group. This suggests that the aerobic-dominant design of our program was insufficient to preserve muscle mass, aligning with current recommendations that stress the importance of including resistance training for sarcopenia prevention [32,33,34]. Future adaptations of this program should therefore incorporate progressive resistance exercises alongside aerobic training.

The reductions in blood pressure were observed in intervention group, it has been suggested that combined aerobic and low-intensity resistance exercise training increases basal nitric oxide production and decreases arterial stiffness even without body weight reduction in older adult [35].

Both improvements, in BB and HbA1c achieved are clinically meaningful, corresponding to a 20–40% lower risk of cardiovascular and microvascular events. In institutionalized elderly populations, such improvements can reduce polypharmacy, healthcare utilization, and disability. Importantly, the intervention was feasible in low-resource settings, requiring only minimal equipment and leveraging existing community infrastructure.

Strengths include being one of the first large-scale studies in Latin American elderly welfare homes, with a full year of follow-up and monthly monitoring. High adherence and standardized measurement protocols strengthen internal validity.

Regarding results, a potential limitation involves the observed discrepancy between the blood pressure exclusion criteria (≥140/90 mmHg) and the reported baseline median (148/92 mmHg). It is important to clarify that the exclusion threshold was applied during the initial screening phase to ensure participant safety and pharmacological stability prior to enrollment. However, the baseline measurements, recorded on the first day of the intervention, reflect intrinsic physiological variability and the potential influence of ‘white coat hypertension’ in an institutional setting. These fluctuations are common in elderly populations with long-standing hypertension and do not compromise the study’s internal validity, as the primary focus was the longitudinal change in pressure relative to each participant’s starting point [36,37].

Other limitation of this study is the heterogeneity observed in the baseline characteristics of the sample with regard to body weight and body mass index (BMI). The intervention group was found to have a significantly higher absolute weight but a lower BMI compared to the control group. Although these variables were considered in the statistical adjustment, initial anthropometric variability should be taken into account when generalizing the body composition results.

Some other limitations also include the design and the lack of dietary and medication adherence data. Due to these limitations in covariate depth, our findings should be interpreted with caution regarding direct causality. The potential for unmeasured confounding remains, and the results are best understood as identifying key trends and generating hypotheses that require validation through future prospective studies with more expansive variable sets. Finally, the predominance of female participants reduces generalizability to men. Future directions include replication in randomized controlled trials, with integration of resistance exercise, nutritional strategies, and longer follow-up.

## 5. Conclusions

A structured, supervised exercise program embedded in government-supported shelters significantly improved blood pressure, glycemic control, and body composition in elderly adults with hypertension and diabetes, beyond pharmacological treatment alone. These findings support integrating low-cost, scalable physical activity programs into standard geriatric care, particularly in resource-limited institutional settings.

## Figures and Tables

**Table 1:** Baseline measures of elderly individuals per group.

Variable	Total n = 260	Control Group n = 129	Intervention Group n = 131	*p* Value
Age (years)	70.0 (66.0–76.0)	71.0 (67.0–77.0)	69.0 (64.0–75.0)	0.009 *
Weight (Kg)	73.5 (67.7–84.4)	70.1 (63.7–78.4)	76.1 (70.7–81.8)	0.001 ***
Height (M)	1.50 (1.47–1.57)	1.57 (1.50–1.60)	1.48 (1.45–1.50)	0.001 ***
Lean Mass (%)	30.2 (28.4–32.1)	29.4 (27.4–33.1)	30.5 (29.6–31.7)	0.054
Fat Mass (%)	37.3 (35.1–38.9)	37.5 (35.7–39.4)	36.5 (34.4–38.6)	0.015 *
Comorbidities				
Hypercholesterolemia (%)	54.6 (142)	53.4 (70)	55.8 (72)	0.710
Hypertriglyceridemia (%)	48.8 (127)	46.6 (51)	51.2 (66)	0.535
Clinic Assessments				
BMI (Kg/$m^{2}$)	31.2 (29.5–34.6)	33.1 (29.0–36.1)	30.8 (29.7–32.6)	0.011 *
SBP (mmHg)	148.0 (140.0–155.0)	148.0 (140.0–155.0)	148.0 (141.0–155.0)	0.562
DBP (mmHg)	90.0 (88.5–94.0)	90.0 (85.0–90.0)	92.0 (90.0–95.0)	0.001 ***
HbA1C (%)	8.5 (8.1–8.8)	8.3 (8.0–8.6)	8.60 (8.3–8.8)	0.001 ***

SBP: Systolic Blood Pressure; DBP: Diastolic Blood Pressure; BMI: Body Mass Index; HbA1C: Glycated Hemoglobin. * *p* ˂ 0.05; *** *p* ˂ 0.001; reports by Mann-Withney U for independent samples at quantitative variables and X^2^ for those qualitative variables.

## Data Availability

The data presented in this study are available on request from the corresponding author due to ethical restrictions. Access will be granted to qualified researchers upon reasonable request and subject to a formal data use agreement with National System for the Integral Development of Families DIF Unidad Central, Huixquilucan.

## Abbreviations

The following abbreviations are used in this manuscript:

BP	Blood Pressure
HbA1c	Glycated Hemoglobin
BMI	Body Mass Index
SNDIF/DIF	National System for the Integral Development of Families
